# Rapid Skin Responses to a Low Humidity Environment and the Benefits of a Fine Fiber Film

**DOI:** 10.1111/srt.70398

**Published:** 2026-10-30

**Authors:** Junko Ishikawa, Akane Kawamoto, Urara Yokose, Noriyasu Ota, Takatoshi Murase

**Affiliations:** ^1^ Biological Science Research Kao Corporation Ichikai‐machi Tochigi Japan

**Keywords:** fine fiber film, low humidity environment, short single stay, stratum corneum, skin response

## Abstract

**Background:**

A short single low‐humidity stay (SSLHS) induces rapid changes in stratum corneum (SC) function, but their significance and inter‐individual variability remain unclear. We examined associations between baseline SC function and rapid SSLHS responses and evaluated whether a lotion‐free fine fiber (FF) film modulates these responses.

**Materials and Methods:**

Forty healthy Japanese women (*n* = 40) completed 6‐h stays at 60% RH and, on the next day, 20% RH (23°C). TEWL, capacitance, and skin texture were measured on the open cheek at 0, 3, and 6 h. During SSLHS, an FF film was applied to the right half of the face and assessed at 0 and 6 h. Pearson's r and within‐subject differences in absolute change (absΔ; FF − open) are reported with 95% confidence intervals (95%CI).

**Results:**

TEWL decreased under both RH conditions, whereas capacitance increased only at 60% RH. Under SSLHS, absΔTEWL (3 h–0 h) was associated with baseline TEWL (*r* = 0.661, 95%CI 0.44–0.81), capacitance (*r* = −0.504, 95%CI −0.70 to −0.23), and SEr (*r* = 0.518, 95%CI 0.25–0.71). During SSLHS, the FF film reduced absΔTEWL (mean difference −1.22 [95%CI −2.36, −0.09]) and increased absΔCapacitance (+2.20 [0.87, 3.54]) versus the open side. Exploratory subgroup analysis suggested larger differences in subjects classified as sensitive.

**Conclusion:**

Rapid low‐humidity responses vary with baseline SC function, and an FF film without lotion modulates short‐term responses to low humidity.

## 1 Introduction

Low‐humidity environments are a common environmental stressor for facial skin and have been associated with rapid changes in stratum corneum (SC) functional parameters and skin appearance. Previous studies reported that even a short exposure (e.g., 6 h) to low relative humidity can alter transepidermal water loss (TEWL), SC hydration, and fine wrinkles in humans, yet the significance of these rapid changes remains unclear—whether they reflect adaptive homeostatic responses or impaired skin adaptability to humidity stress [1, 2, 3].

Importantly, skin responses to the same environmental condition can vary markedly among individuals. Clarifying how baseline SC function relates to the magnitude of rapid responses may help identify phenotypes prone to low‐humidity sensitivity and provide a basis for targeted protective strategies.

Fine fiber (FF) films are thin, flexible membranes that adhere well to the skin and can form a uniform surface coating [4]. Prior work suggested that FF films combined with a water‐based lotion may improve dry skin conditions with the protein expressional changes;[4, 5] however, the effect of an FF film alone on rapid skin responses during a short single low‐humidity stay (SSLHS) has not been established.

Therefore, this study aimed (1) to characterize human facial skin responses to a short, single exposure to low humidity, (2) to examine the relationship between inter‐individual differences in responses and baseline SC functional parameters, and (3) to evaluate whether an FF film without lotion mitigates these responses during low‐humidity exposure.

## 2 Materials and Methods

### 2.1 Ethics

This study was conducted according to the Declaration of Helsinki Principles, and the protocol was approved by the Human Research Ethics Committee of the Kao Corporation (T266‐191015). Informed consent to participate in this study was obtained from each subject after the procedures had been explained. The study was prospectively registered in the University Hospital Medical Information Network Clinical Trials Registry (UMIN‐CTR; UMIN000038552) on December 1, 2019, before initiation of the study. The approved study protocol has been retained and is available from the corresponding author upon reasonable request. No amendments to the approved protocol were made between ethics approval and the conduct of the study.

### 2.2 Study Design and Setting

This was a single‐center, open‐label, non‐randomized, split‐face study. On Day 2 (20% RH), the FF film was applied to the right half of the face in all participants, while the left half served as the open control. The treatment side was fixed and was not randomized. Because the FF film was visible on the skin, blinding of participants was not feasible. The assessors performing the biophysical measurements were also not blinded to the treatment side and were aware that the FF film had been applied to the right side of the face.

Two consecutive‐day, within‐subject, comparison:

Day 1: SSMHS (60% RH, 6 h)

Day 2: SSLHS (20% RH, 6 h)

Split‐face intervention on Day 2: FF film applied to right of the face; the other half left open.

The examinations were conducted on two consecutive days, from December 2019 to February 2020 in Tochigi, Japan. The examination schedule is shown in Figure 1.

**FIGURE 1:**
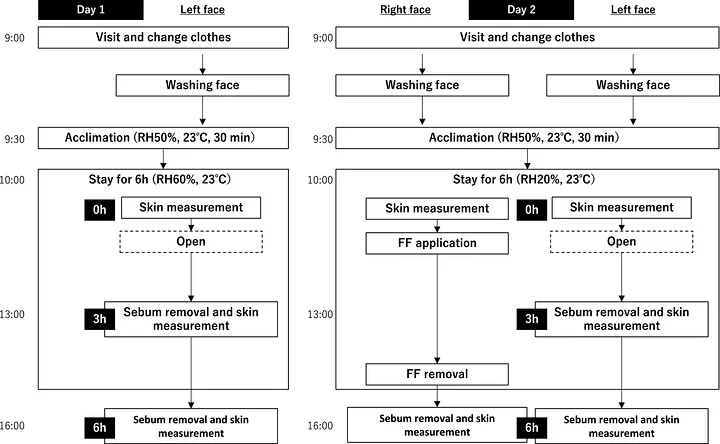
Scheme showing the protocol of this study. Forty Japanese female subjects with normal skin (21 ‐ 59 years old; mean, 39) were enrolled and examinations were conducted on two consecutive days. The subjects stayed at 60% RH (23°C, 6 h, Day 1) and at 20% RH (23°C, 6 h, Day 2). The FF membrane covered the right side of the face for 6 h after the 0 h measurement on Day 2. RH; relative humidity.

Based on the sample size calculation for the primary correlation and the primary paired comparison, at least 37 participants were required; therefore, 40 participants were enrolled.

### 2.3 Participants

Forty healthy Japanese women aged 20–59 years were recruited from the local area (Tochigi, Japan) (Table 1). All participants provided written informed consent prior to any study procedures. Participants were excluded if they were pregnant or breastfeeding; had facial skin diseases (e.g., atopic dermatitis, psoriasis, or severe acne) or marked sunburn at the assessment site; were undergoing or had recently undergone cosmetic/dermatological procedures (e.g., chemical peels or laser treatment); were using medications or receiving medical treatment that could affect skin condition; were participating in other interventional studies; or were unable/unwilling to comply with study restrictions and procedures.

**TABLE 1:** Participants Forty Japanese female subjects with healthy skin (age range, 21 – 59 years; mean age: 39 years) were enrolled in the study. The group consisted of 16 sensitive subjects, 16 non‐sensitive subjects, and 8 unclassified subjects. There were no missing or excluded values.

Characteristic	Value
Participants included in the analysis, *n*	40
Age, mean (SD)	39 (9.5)
Age range (min–max)	21–59
Sex, *n* (%)	Female, 40 (100)
Sensitive group, *n* (%)	16 (40)
Insensitive group, *n* (%)	16 (40)
Non‐classified group, *n* (%)	8 (20)
Missing data, *n* (%)	0 (0)

### 2.4 Procedures

Each subject changed into designated clothes to eliminate external influence, and then entered an acclimation room (50 ± 5% RH, 23 ± 2°C). After washing her face, each subject acclimated for 30 min on both days. On Day 1, each subject stayed in a medium humidity room (60 ± 5% RH, 23 ± 2°C) for 6 h. Skin measurements were conducted on the left side of their face at 0, 3 and 6 h. Sebum was removed with an oil blotting film at 3 and 6 h prior to measurements. On Day 2, each subject entered an acclimation room (50 ± 5% RH, 23 ± 2°C), before moving to a low humidity room (20 ± 5% RH, 23 ± 2°C) for 6 h. Skin measurements were conducted on the left side of their face, specifically on the cheek, at 0, 3 and 6 h. Sebum was removed from the left side of their face at 3 and 6 h before measurements.

### 2.5 Intervention: FF Film

A FF film was applied only to the right half of the face immediately after the skin measurement at 0 h on Day2. On the FF‐treated side of the face, skin measurements were conducted on the cheek at 0 h (before the FF film application) and at 6 h (after the FF film application, removal and sebum removal). A FF film was applied to each subject using a portable device (Kao Corporation, Tokyo, Japan) [4] and remained on the skin surface without any lotion application for 6 h.

### 2.6 Outcomes / Measurements

TEWL values were measured once for each site using an AquaFlux (AF200, Biox Systems Ltd, London, UK). SC hydration was measured five times for each site using a Corneometer CM 825 (Courage + Khazaka electronic GmbH, Köln, Germany) as capacitance, and the average value was calculated. Skin surface texture was assessed once for each site using a Visioscan VC 98 (Courage+Khazaka), and four Skin Evaluation of Living Skin (SELS) parameters were obtained: Ser (roughness), SEsc (scaliness), SEsm (smoothness), and SEw (wrinkles) using SELS 2000 software (Courage+Khazaka).

Primary endpoint (the correlation between cutaneous responses and initial SC function) was absolute delta TEWL (absΔTEWL) (3 h‐0 h) on open side at 20 ± 5% RH, 23 ± 2°C stay (SSLHS).

Primary endpoint (the efficacy of FF film) was left‐right difference (FF‐Open) of absolute delta TEWL (absΔTEWL) (6 h‐0 h).

### 2.7 Derived Variables

Delta (Δ) values were calculated as the raw value at each time point subtracted from the raw value at 0 h.

Absolute delta (absΔ) values were derived from each delta (Δ) value.

Effect of FF was left‐right difference of absΔ TEWL(6 h‐0 h) in FF site minus absΔ TEWL(6 h‐0 h) in open site.

Group assignment: Subjects were categorized based on their absΔTEWL(3 h‐0 h). Specifically, 40% of the subjects exhibiting the highest absΔTEWL values (≥3.4 g/m^2^/h) were designated as the sensitive group, while 40% of subjects with the lowest absΔTEWL values (≤1.2 g/m^2^/h) were classified as the insensitive group. The remaining 20% of subjects were categorized as the non‐classified group (1.2< absΔTEWL < 3.4 g/m^2^/h). This subgroup classification was exploratory and data‐derived and was not a prespecified or independently validated definition of skin sensitivity. Accordingly, all analyses stratified by the sensitive and insensitive groups were considered exploratory.

### 2.8 Statistical Analysis

Analyses were conducted using a complete‐case approach, and the sample size (n) is reported for each analysis. Descriptive data are presented as mean (SD). Associations between baseline SC parameters and response magnitude were evaluated using Pearson's correlation coefficients with 95% confidence intervals calculated using Fisher's z transformation. Within‐subject comparisons between the FF‐treated and open sides are presented as mean paired differences with 95% confidence intervals.

The analyses specified as primary endpoints were treated as primary analyses. Analyses stratified by the data‐derived sensitive and insensitive groups were considered exploratory. For the subgroup analyses, the within‐subject difference in absolute change between the FF‐treated and open sides was tested against zero. To account for multiple testing, p‐values for the four subgroup comparisons comprising two outcomes (TEWL and capacitance) in the two subgroups (sensitive and insensitive) were adjusted using the Holm method. Both unadjusted and Holm‐adjusted p‐values are reported. All statistical tests were two‐sided.

Analyses were performed using Microsoft Excel (Microsoft 365, Microsoft Corporation, Redmond, WA, USA).

### 2.9 Sample Size Justification

Primary analysis (correlation): using absΔTEWL3 h‐0 h and baseline capacitance as the primary correlates, with a minimum correlation coefficient of |r| = 0.45, a two‐sided α of 0.05, and a power of 0.8, the sample size was calculated using Fisher's Z‐table to be 37 cases; anticipating missing values and exclusions, the sample size was set to *n* = 40.

Primary Analysis (Between‐sites): For the paired analysis comparing the presence and absence of FF, to ensure a difference of 1.0 (absΔTEWL6 h‐0 h) between the FF and open sites, we used the SD of 1.81 for the difference in absΔTEWL3 h‐0 h from the pilot study (due to a maximum measurement time of 3 h). To detect a clinically meaningful between‐group difference of 1, with a two‐sided *α* = 0.05, and a power of 0.80, the required sample size was calculated as *n* = 26; we estimated 30 to account for additional variability. We enrolled 40 subjects, exceeding the required sample size.

## 3 Results

### 3.1 Participants

Forty healthy Japanese female subjects were included in the analyses (*n* = 40; age, 39 ± 9.5, 21–59). No participants were excluded after enrollment.

For the exploratory subgroup analyses, participants were categorized into sensitive (*n* = 16), insensitive (*n* = 16), and non‐classified (*n* = 8) groups based on absΔTEWL (3 h–0 h) at 20% RH, as describes in the Materials and Methods (Table 1).

### 3.2 Raw SC Parameters at Baseline and Follow‐Up (Open Side, 20%RH and 60%RH)

Raw values of TEWL, capacitance, and skin surface texture parameters at 0 h, 3 h, and 6 h under 20% and 60% RH are summarized in Table 2. TEWL decreased over time under both RH conditions, whereas capacitance increased at 60% RH but showed minimal change at 20% RH (Table 2).

**TABLE 2:** Values are presented as mean (SD). Measurements were obtained on the open side of the face.

Parameter	Unit	20%RH 0 h	20%RH 3 h	20%RH 6 h	60%RH 0 h	60%RH 3 h	60%RH 6 h	n
TEWL	g/m^2^/h	26 (8.7)	24 (6.8)	23 (6.7)	27 (7.1)	26 (8.1)	24 (6.4)	40
Capacitance	A.U.	58 (11)	60 (9.6)	59 (9.5)	59 (11)	63 (9.8)	64 (9.4)	40
SEr	A.U.	3.3 (1.7)	3.2 (1.6)	3.1 (1.6)	3.3 (1.6)	3.2 (1.5)	3.1 (1.5)	40
SEsc	A.U.	0.9 (0.3)	0.9 (0.2)	0.8 (0.2)	0.9 (0.4)	0.8 (0.3)	0.8 (0.2)	40
SEsm	A.U.	33 (4.6)	34 (4.8)	35 (5.2)	33 (4.3)	36 (4.9)	36 (4.9)	40
SEw	A.U.	38 (3.4)	38 (2.8)	37 (2.7)	38 (3.5)	38 (3.0)	38 (2.4)	40

### 3.3 Main Correlations (Baseline vs Response Magnitude at 20%RH)

absΔTEWL (3 h–0 h) on the open side was positively associated with baseline TEWL and SEr and negatively associated with baseline capacitance (Table 3; Figure 2).

**TABLE 3:** Pearson's correlation coefficient (r) is shown with 95% confidence intervals (95%CI) calculated using Fisher's z transformation. absΔTEWL (3 h–0 h) indicates the absolute change in TEWL between 0 and 3 h during the 20% RH exposure on the open side, computed as (|TEWL_{3 h}—TEWL_{0 h}|). TEWL, transepidermal water loss; RH, relative humidity.

Outcome	Baseline predictor	r	95%CI	n
absΔTEWL (3 h–0 h), 20%RH (open)	TEWL (0 h)	0.661	0.44 to 0.81	40
absΔTEWL (3 h–0 h), 20%RH (open)	Capacitance (0 h)	−0.504	−0.70 to ‐0.23	40
absΔTEWL (3 h–0 h), 20%RH (open)	SEr (0 h)	0.518	0.25 to 0.71	40

**FIGURE 2:**
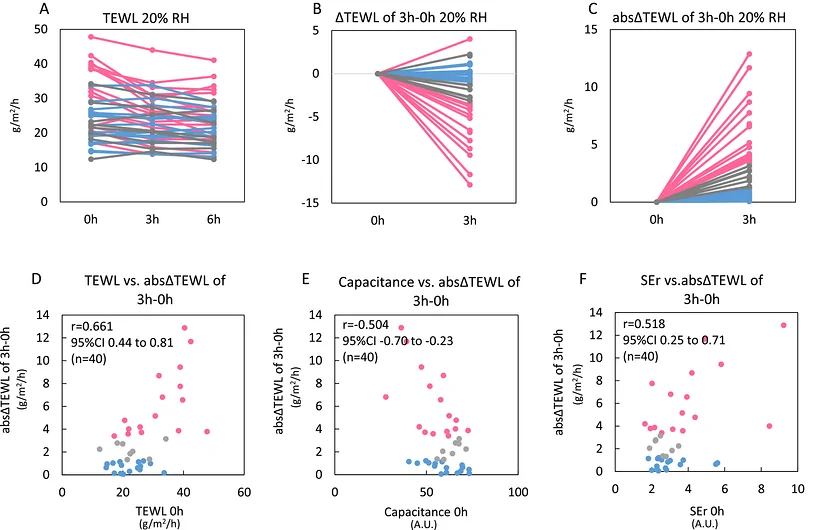
Individual variation of (A) raw values of TEWL, (B) changed values from 0 h (ΔTEWL (3 h‐0 h)) and (C) absolute change values from 0 h (absΔTEWL(3 h‐0 h)) during a 3 h stay at 20% RH. 40% of subjects with upper absΔTEWL values were set as the sensitive group (red, *n* = 16), 40% of subjects with lower absΔTEWL values were set as the insensitive group (blue, *n* = 16) and the other 20% of subjects were set as the non‐classified group (gray, *n* = 8). Pearson's correlation coefficient (r) is shown with 95% confidence intervals (95%CI) calculated using Fisher's z transformation. Pearson's correlations between absΔTEWL values and SC functional parameters at 0 h are shown, such as (D) TEWL, (E) Capacitance, and (F) SEr. RH; relative humidity.

### 3.4 Effects of FF Film (Raw Values at 0 and 6 h at 20%RH and Within‐Subject Difference in absΔ(FF‐Open))

Raw values at 0 and 6 h on the open and FF‐treated sides are shown in Table 4. At 6 h, TEWL did not decrease and capacitance increased more on the FF‐treated side compared with the open side (Table 4). Within‐subject difference in absΔ between sides, that of TEWL was minus, but that of capacitance was plus (Table 4, Figure 3).

**TABLE 4:** Values are presented as mean (SD). Measurements were obtained at 0 h (baseline) and 6 h during the 20% RH exposure on the open and FF‐treated sides. (ΔX) denotes the change from 0 to 6 h (ΔX = X_{6 h}—X_{0 h}), and (absΔX) denotes the absolute change (absΔX = |ΔX| = |X_{6 h}—X_{0 h}|). The rightmost column summarizes the within‐subject difference in absolute change between sides, defined as (absΔX_{FF}—AbsΔX_{Open}), and is reported as the mean paired difference with 95% confidence intervals (95%CI). TEWL, transepidermal water loss; RH, relative humidity; FF, fine fiber.

Parameter	Unit	Open 0 h	Open 6 h	FF 0 h	FF 6 h	Within‐subject difference in absΔ (FF − Open), mean [95%CI]	n
TEWL	g/m^2^/h	26 (8.7)	23 (6.7)	27 (7.8)	25 (6.5)	−1.22 [‐2.36, ‐0.09]	40
Capacitance	A.U.	58 (11)	59 (9.5)	55 (12)	59 (9.9)	2.20 [0.87, 3.54]	40

**FIGURE 3:**
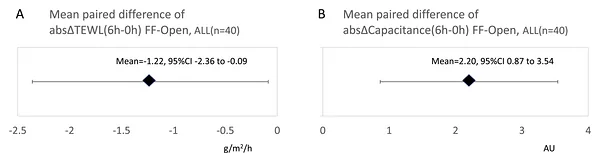
Effects of the FF film on the magnitude of skin responses at 20% RH (*n* = 40). Points indicate mean within‐subject differences in absolute change from 0 to 6 h (absΔ; FF − open), and error bars indicate 95% confidence intervals. (A) absΔTEWL; Negative value indicates a smaller magnitude of change on the FF‐treated side. (B) absΔCapacitance; Positive value indicates a larger magnitude of change on the FF‐treated side.

### 3.5 Exploratory Analysis of FF Effects in Sensitive and Insensitive Subgroups

Raw values at 0 and 6 h stratified by sensitivity group are summarized in Table 5. In the exploratory subgroup analyses, the sensitive group showed a smaller magnitude of change in TEWL and a larger magnitude of change capacitance on the FF‐treated side than on the open side. These differences remained statistically significant after Holm adjustment for the four subgroup comparisons (Table 6; Figure 4). In contrast, the corresponding differences in the insensitive group were not statistically significant after Holm adjustment. In the sensitive group, the mean within‐subject difference in absΔTEWL (FF − open) was −3.13 (95% CI, −5.47 to −0.79; unadjusted *p* = 0.012; Holm‐adjusted *p* = 0.036), and that in absΔCapacitance was 4.11 (95% CI, 1.71 to 6.52; unadjusted *p* = 0.0024; Holm‐adjusted *p* = 0.0096).

**TABLE 5:** Values are presented as mean (SD). Measurements were obtained at 0 h (baseline) and 6 h during the 20% RH exposure on the open and FF‐treated sides. Sensitivity groups were defined based on absΔTEWL (3 h–0 h) on the open side at 20% RH (sensitive: ≥ 3.4 g/m^2^/h, *n* = 16; insensitive: ≤ 1.2 g/m^2^/h, *n* = 16). TEWL, transepidermal water loss; RH, relative humidity; FF, fine fiber. Participants in the non‐classified group (1.2 < absΔTEWL (3 h–0 h) < 3.4 g/m^2^/h; *n* = 8) are not shown in this table. The subgroup classification and all analyses stratified by sensitivity group were exploratory.

Group	Side	TEWL 0 h mean (SD)	TEWL 6 h mean (SD)	Capacitance 0 h mean (SD)	Capacitance 6 h mean (SD)	n
Sensitive	Open	32 (9.1)	25 (7.9)	54 (12)	55 (9.5)	16
Sensitive	FF	30 (9.9)	27 (8.1)	52 (13)	59 (10)	16
Insensitive	Open	22 (5.4)	21 (4.8)	60 (10)	61 (10)	16
Insensitive	FF	24 (5.5)	23 (4.9)	56 (12)	60 (12)	16

**TABLE 6:** Exploratory subgroup analyses of FF effects after adjustment for multiple comparisons.

Group	Outcome	Mean difference (FF − Open)	95% CI	Unadjusted p	Holm‐adjusted p
Sensitive	absΔTEWL	−3.13	−5.47 to −0.79	0.012	0.036
Sensitive	absΔCapacitance	4.11	1.71 to 6.52	0.0024	0.0096
Insensitive	absΔTEWL	0.70	−0.45 to 1.85	0.212	0.424
Insensitive	absΔCapacitance	1.08	−1.00 to 3.17	0.285	0.424

**FIGURE 4:**
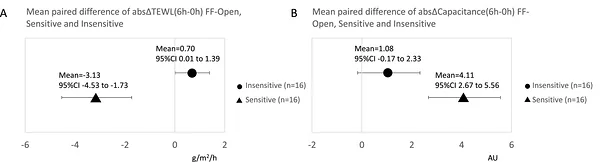
Exploratory analysis of FF film effect stratified by sensitivity group　at 20% RH. Mean within‐subject differences in absΔ (FF − open) are shown with 95% confidence intervals for the sensitive (*n* = 16) and insensitive (*n* = 16) groups. (A) absΔTEWL; a negative value indicates a smaller magnitude of change on the FF‐treated side. (B) absΔCapacitance; a positive value indicates a larger magnitude of change on the FF‐treated side. P‐values for the four subgroup comparisons were adjusted for multiple testing using the Holm method; unadjusted and adjusted p‐values are reported in Table 6.

## 4 Discussion

This study investigated rapid facial skin responses to a short single stay under low humidity and explored factors associated with inter‐individual variability, as well as the potential of a FF film (without lotion) to modulate these responses. The main findings were: (i) TEWL decreased over time under both 20% and 60% RH, whereas capacitance increased at 60% RH but showed minimal change at 20% RH (Table 2); (ii) the magnitude of the rapid TEWL response under 20% RH, quantified as absΔTEWL (3 h–0 h), was associated with baseline SC functional parameters (baseline TEWL, capacitance, and SEr; Table 3, Figure 2); and (iii) at 20% RH, the FF‐treated side showed higher capacitance at 6 h than the open side, with smaller differences in TEWL also observed between sides, particularly in the sensitive subgroup (Tables 4, 5; Figures 3, 4).

### 4.1 Rapid SC Responses During Short‐Term Humidity Exposure

A decrease in TEWL was observed during the 6‐h stay under both RH conditions (Table 2). Although low humidity is generally considered a stressor that can impair SC condition, rapid TEWL reductions during short exposures have been reported previously and remain mechanistically unresolved [2, 3]. Importantly, TEWL reflects not only barrier properties but also dynamic physiological and environmental influences (e.g., skin surface microclimate and perfusion). Thus, the observed TEWL decrease during short stays may represent a transient adaptive response rather than a straightforward indicator of improved barrier function. In contrast, capacitance increased at 60% RH but remained largely unchanged at 20% RH (Table 2), supporting the expected influence of ambient humidity on SC hydration over a short timeframe.

### 4.2 Baseline SC Function and Inter‐Individual Variability in Low‐Humidity Response

A key aim of this study was to link inter‐individual variability in low‐humidity response to baseline SC function. Under 20% RH, absΔTEWL (3 h–0 h) on the open side was positively associated with baseline TEWL and SEr and negatively associated with baseline capacitance (Table 3; Figure 2). These associations suggest that individuals with comparatively lower SC hydration and rougher surface characteristics at baseline may exhibit larger short‐term fluctuations in TEWL when exposed to low humidity. From a practical perspective, absΔTEWL (3 h–0 h) may serve as an informative response metric for characterizing inter‐individual variability in rapid responses to humidity changes. In the present study, grouping based on this metric was used as an exploratory framework to investigate such variability. However, because the groups were derived from the observed early TEWL response itself, this classification should not be considered an independently validated phenotype of low‐humidity sensitivity.

### 4.3 FF Film Effects During Low‐Humidity Exposure

The FF film was evaluated as a lotion‐free, short‐term protective approach during the 20% RH stay. At 6 h, capacitance was higher on the FF‐treated side than on the open side (Table 4; Figure 3), indicating that the FF film can promote or preserve SC hydration under low humidity. Differences in TEWL between sides were also observed at 6 h (Table 4). Exploratory subgroup analyses suggested that the FF‐related differences in TEWL and capacitance were more pronounced in subjects classified as sensitive, and these differences remained statistically significant after Holm adjustment for multiple comparisons (Table 6; Figure 4). However, because the sensitivity groups were defined using the early TEWL response itself, these subgroup findings should be considered hypothesis‐generating rather than confirmatory. These findings are consistent with the concept that a thin, adherent film can alter the skin surface microenvironment (e.g., by moderating water vapor exchange) like a simulated SC, thereby supporting hydration under dry conditions. Prior reports have shown that FF films combined with lotion can improve dry skin with protein levels for one week, [4, 5] but the present results extend this evidence by suggesting that the film alone can influence short‐term biophysical responses during a brief low‐humidity exposure.

### 4.4 Limitations and Future Directions

Several limitations should be noted. First, the study used an open‐label, non‐randomized split‐face design, and the FF film was applied to the right side of the face in all participants. Therefore, inherent left–right asymmetry in facial skin properties or environmental responses cannot be excluded, and part of the observed between‐side differences may reflect side‐related effects rather than the FF film itself. In addition, the assessors performing the biophysical measurements were not blinded to the treatment side; therefore, measurement bias cannot be completely excluded.

Second, the order of humidity exposure was fixed, with 60% RH on Day 1 followed by 20% RH on Day 2. Thus, the effects of humidity cannot be fully distinguished from day effects or possible carryover effects from the preceding exposure. Comparisons between the two humidity conditions should therefore be interpreted cautiously. Future studies should use randomized or counterbalanced exposure order with an adequate washout period.

Third, the sensitive and insensitive groups were defined using absΔTEWL (3 h–0 h), and subsequent analyses of TEWL‐related responses within these data‐derived groups are not fully independent of the grouping variable. Therefore, circularity, regression to the mean, and inflation of apparent subgroup differences cannot be excluded. Although multiplicity was addressed using the Holm method, these subgroup analyses remain exploratory and hypothesis‐generating and require confirmation in an independent cohort using prespecified and independently defined sensitivity criteria.

Fourth, the study population consisted of healthy Japanese women; therefore, generalizability to other populations, males, or individuals with dermatological conditions or independently defined sensitive‐skin phenotypes requires further investigation. Finally, only biophysical measurements were assessed, and potential molecular or physiological mediators were not examined (e.g., differentiation markers, inflammatory markers, neuroendocrine stress‐related pathways, or skin blood flow) [5, 6, 7]. Future randomized studies incorporating additional biomarkers and longer or repeated exposures may help clarify mechanisms underlying rapid TEWL fluctuations and identify individuals who may benefit most from film‐based protection.

## 5 Conclusion

In conclusion, short‐term humidity exposure induced measurable changes in SC functional parameters, and the magnitude of the rapid TEWL response under low humidity was associated with baseline SC function. Moreover, an FF film without lotion increased SC hydration and modulated short‐term responses during low‐humidity exposure. Exploratory subgroup analyses suggested differences according to the early TEWL response; however, these findings require confirmation using prespecified and independently defined sensitivity criteria.

## Conflicts of Interest

All authors are employees of Kao Corporation. Kao Corporation funded the study and provided the fine fiber film and the device used for its application.

## Funding Information

This study was funded by Kao Corporation. The fine fiber film and the device used for its application were provided by Kao Corporation.

## Data Availability

The data of this study are available from the corresponding author upon reasonable request.
